# Who Did I Swipe On? Accuracy and Self-Presentation in Online Dating

**DOI:** 10.1177/01461672251386488

**Published:** 2025-11-13

**Authors:** Sarra Jiwa, Norhan Elsaadawy, Erika N. Carlson

**Affiliations:** 1 University of Toronto, Mississauga, Ontario, Canada; 2 Carleton University, Ottawa, Ontario, Canada

**Keywords:** accuracy, personality, online dating, self-presentation, dating, person perception, individual differences, impression formation, impression management, romantic relationships

## Abstract

Many people use online dating profiles to meet partners and screen potential dates. Unlike other online contexts, targets might be more motivated to misrepresent their personality, making accuracy difficult. How strongly are people motivated to misrepresent themselves, how transparent is personality, and which individual differences might explain these processes? Online daters (targets, *N* = 180) submitted their profiles, described their personality and the impression they wanted to convey. Judges (*N* = 196) viewed these profiles and rated targets’ personalities. Overall, targets wanted to be seen accurately and positively, and they successfully presented desired personas without their personality leaking through, suggesting being seen accurately is within targets’ control. Some processes were related to outcomes (e.g., swiping decisions) and explained by individual differences (e.g., attachment). These findings highlight the importance of considering self-presentational goals in online dating and when indexing accuracy in general.

Personality is an important consideration when deciding who to date (Fiore et al., 2008; Klohnen & Luo, 2003). Given that online dating is becoming the most common way to meet romantic partners (Rosenfeld et al., 2019) and browsing and swiping on profiles is necessary to facilitate a date (Finkel et al., 2012), online daters try to convey their personality while also making a positive impression (Ellison et al., 2006). Importantly, picking the right strategy and implementing it effectively has critical implications for their dating success, namely whether judges select their profile, and consequently, whether they can meet their broader relationship goals (e.g., going on a date, finding a partner).

Given the growing use of online dating, it is important to know if profile makers (targets) can present their desired impression, but also if online daters can accurately judge the personalities of the people whose profiles they come across, regardless of the target’s motives. While accuracy in online mediums (i.e., social media; Back et al., 2010; Human & Biesanz, 2013; Human et al., 2021; Orehek & Human, 2017; Osterholz et al., 2023; Stopfer et al., 2014) and in-person romantic interactions (Kerr et al., 2020) has been studied previously, online dating introduces a theoretically meaningful context to explore accuracy due to the combination of targets’ strong self-presentational motives and ability to curate cues.

The current study tests the degree to which people: (a) want to present themselves accurately or differently on their dating profiles, (b) are seen accurately and/or in line with how they wanted to be seen, and (c) how these strategies and processes are related to dating success (i.e., being chosen by daters). We also explore key moderators to better understand who tends to be seen accurately or in line with their self-presentational goals.

## How Do People Want to Be Seen in Online Dating?

Impression management is especially important in dating because, from an evolutionary standpoint, finding a mate is a primary social motivation (Schaller et al., 2017). In online dating in particular, impression management is at the forefront given that reactions to a target’s profile directly influence how likely the target is to reach their broader relationship goals (e.g., find a date or romantic partner).

On one hand, people might want to present themselves accurately to attract compatible partners, especially if they have goals to meet a suitable match (e.g., Tom wants someone who will like him for him). Likewise, the anticipation of meeting someone in person might motivate people to present themselves accurately to avoid disappointing their dates and experiencing rejection down the line (Gibbs et al., 2006; Walther, 1996; Whitty, 2008).

On the other hand, people might be motivated to present a self that is not fully accurate to attract the best potential dates but also to fulfill needs for worthiness and self-esteem (Leary & Kowalski, 1990). Indeed, people misrepresent themselves on characteristics that are important to potential partners (e.g., height or weight; Toma et al., 2008) to increase their perceived desirability, a pattern that likely extends to personality traits (Barclay, 2010; Schaffhuser et al., 2014). Thus, people might emphasize some aspects of their personality and/or omit others to make a particular type of impression (e.g., Tom presents himself as carefree, despite being quite rigid). In sum, while daters report a desire to present themselves accurately and differently (Ellison et al., 2006), it is unclear how they actually try to present themselves—a question we aim to address here.

## Are People Seen Accurately and/or in Line With Their Self-Presentational Goals?

People might approach online dating with motivations to be seen accurately or differently from who they are, but are they able to achieve these goals? Past work has demonstrated that people are seen accurately across contexts with minimal information, such as a photo (Kaurin et al., 2018) or email address (Back et al., 2008), and in contexts where they can curate cues, such as social media (Back et al., 2010; Human & Biesanz, 2013; Human et al., 2021; Orehek & Human, 2017; Osterholz et al., 2023; Stopfer et al., 2014). As such, we will test *accuracy—*the degree to which targets are seen as they see themselves*—*in online dating to determine if this context shows similar effects.

Notably, the strong motivation to omit and/or emphasize certain traits more than others in online dating might obscure accuracy. Not only do people face rejection if they fail to make the right impression, but online mediums specifically allow people the unique opportunity to convey particular impressions by curating cues on their profile (Hall et al., 2014; Stopfer et al., 2014), so it is important to know if online daters can take advantage of this medium to make their desired impressions. As such, we first explore whether people achieve *self-presentational success*—the degree to which people are seen in line with their self-presentational goals, which could include being transparent or being seen in ways that differ from one’s global self-view.

When people want to present a different self online, are they successful and/or does their personality leak out? Building on our test of self-presentational success, we explore the degree to which people successfully portray an impression that differs from their self-views (e.g., Tom intentionally curates a profile to make it seem like he is more organized than he really is), an ability we call *skilled misrepresentation*. This test will reveal if targets are seen in line with their desired impressions, not because they want to be seen accurately and are, but specifically because they are able to skillfully portray a different self. On social media, people tend to be seen accurately versus in line with their ideal self, but online dating might present a stronger desire to misrepresent the self (Back et al., 2010). Furthermore, building on our test of accuracy, we test if parts of targets’ personalities leak through, even when they do *not* want to be seen accurately, which we call *accidental transparency*. Accidental transparency might occur in online dating because targets may fail to appreciate which cues they are providing via photos or standardized prompts, revealing if being transparent is beyond a target’s control.

Taken together, the current research will reveal (a) how well people’s personalities come across in online dating, (b) if people can achieve their self-presentational goals in online dating—whether those goals are to be seen accurately or not, and (c) if part of the observed accuracy in online dating is intentional or accidental.

## Is Accuracy and/or Self-Presentational Success Desirable?

Given that targets have goals to be seen accurately and/or differently than their true personality, which strategy is successful, and is dating success impacted when targets are unable to achieve their goals?

Daters report preferring genuine profiles versus profiles that signal deception (Ellison et al., 2006; Wotipka & High, 2016), suggesting that being seen accurately should predict liking. Indeed, subjective feelings of understanding another person promote relationship development (Human et al., 2013, 2020), especially online (McKenna et al., 2002). However, in early romantic relationships, being seen positively seems to be more beneficial than being known (Campbell et al., 2006). Indeed, in speed dating, being seen accurately was associated with lower romantic interest (Kerr et al., 2020). So, it might be that daters prefer people who are high in self-presentational success, or someone who is a socially skilled dater. Thus, while daters report liking transparency, transparency as a strategy can backfire.

In the current work, we explore if accuracy as well as self-presentational success predicts romantic interest based on daters’ swiping decisions.^1^ We also consider the degree to which accuracy is intentional versus accidental, which might account for these conflicting predictions for accuracy. Indeed, accuracy might lead to unfavorable outcomes when it is accidental (i.e., judges detect parts of targets’ personalities that they are trying to hide). Notably, if targets are less liked when they are seen accurately but more liked when they successfully misrepresent their personality in online dating, it would suggest that daters have poor knowledge of their preferences (Eastwick & Finkel, 2008).

## Moderators of Accuracy and Self-Presentation

It is likely that people endorse motivations for accuracy and self-presentation to different degrees and that some people are seen more accurately and/or are better at self-presenting. Specifically, we predict that some people will be seen accurately in part because they want to be, whereas others will be seen accurately despite wanting to hide themselves. Here, we outline the following key moderators of these processes: expressiveness, self-esteem, relationship goals, attractiveness, and attachment.

With respect to being seen accurately (and wanting to be), people who are seen accurately tend to be confident in themselves and comfortable displaying their true personality, and as such, share many valid personality cues (i.e., they are expressive; Colvin, 1993; Human & Biesanz, 2013; Human et al., 2019). Thus, we predict people who provide more information on their profiles (i.e., expressive), and those with high self-esteem will want to be, and will be seen, more accurately. Likewise, long-term relationship seekers may want to be seen accurately and curate their profiles accordingly because they want to find compatible mates. Finally, attractive individuals may be more willing to present their personality accurately if they already have expectations of being liked, especially in the dating context. Moreover, attractive individuals already tend to garner more attention from judges (Langlois et al., 2000), facilitating accuracy (Capozzi et al., 2020; Lorenzo et al., 2010). Thus, in addition to wanting to be seen accurately, they are likely seen accurately as well. In sum, those with high self-esteem, informative profiles, those seeking long-term relationships, and attractive targets should want to be seen accurately and curate their profiles in ways that lead to successful self-presentation.

We also expect that some people will want to be seen differently than who they are, but these individuals may vary in their success at skilled misrepresentation. Broadly, we expect insecure attachment to be related to wanting to mask the self. Anxiously attached individuals may want to be seen differently than who they are, and to do so, share less information (or mask or distort cues) out of fear of rejection (Mosley et al., 2020), whereas avoidantly attached daters may not want to self-disclose to avoid intimacy (Gillath et al., 2010; Mikulincer & Nachshon, 1991). However, we expect that anxiously attached individuals will fail at their goal whereas avoidantly attached individuals are more likely to succeed. We expect that anxiously attached individuals will be unsuccessful masking who they are and instead will be more accidentally transparent based on past work showing that anxious attachment can be detected from photographs and in-person dates (Alaei et al., 2020; Tu et al., 2022). Avoidantly attached individuals might be more successful at skilled misrepresentation given that avoidance does not tend to be observable (Tu et al., 2022).

## The Current Study

Overall, we examine how targets approach online dating and whether their strategies are successful. First, we explore self-presentational goals, the degree to which people are seen accurately (including when accuracy is accidental), and the degree to which people achieve their self-presentational goals. Next, we explore how these processes relate to being chosen by judges, which will reveal whether the strategies targets use are successful. Finally, we explore key moderators of these processes, which might help explain what drives them. Together, this work demonstrates how people want to be and are seen in online dating. This study also builds on current accuracy literature by exploring whether accuracy can be achieved when targets have strong motivations to mask parts of the self, if their personality leaks through despite these motivations, and if daters are able to strategically curate their desired impressions.

## Method

The data, R code, and Supplemental Materials and pre-registration for this study can be found on OSF: https://osf.io/4htdc/. We pre-registered questions related to accuracy, but questions involving self-presentation should be considered exploratory. This study was approved by the research ethics board at the University of Toronto, Mississauga. No additional data was collected after obtaining results. The data collection procedures were pre-registered and codebooks for targets https://osf.io/qstfp/ and judges https://osf.io/3xeaq/ are on OSF.

### Participants

Power to detect accuracy and individual differences in targets depends on the number of targets, judges, and judgments made (Biesanz, 2021). We based our sample size on other studies that have used comparable designs (approximately 100 judges and targets; Back et al., 2010; Osterholz et al., 2023).

#### Targets: Online Dating Profiles

Online dating users who were 18 years of age or older and living in Canada or the USA were recruited through social media (e.g., Facebook, Instagram, Reddit), the participant pool at a Canadian university, flyers at various universities, and the lab website. Participants were required to upload a real online dating profile, pass all the attention check items, and provide sensical responses to all open-ended questions to be included. The final sample consisted of 180 targets aged 18 to 30 years old (see Table 1 for demographics). Participants were compensated $15 CAD (or the USD equivalent).^2^

**Table 1.** Sample Characteristics of Targets and Judges.

Sample characteristics	Targets				Judges			
	*N*	%	*M*	*SD*	*N*	%	*M*	*SD*
Age			21.34	2.99			22.19	4.02
Gender								
Woman	124	69.00			96	48.98		
Man	49	27.00			92	46.94		
Non-Binary/Other	6	3.00			6	3.06		
Prefer to self-identify	0	0.00			1	0.51		
Preferred not to disclose	1	0.50			1	0.51		
Ethnicity								
Black	25	13.89			48	25.00		
White	56	31.11			53	27.04		
East Asian	22	12.22			19	9.69		
Latin American	6	3.33			8	4.08		
Indigenous	0	0.00			4	2.04		
Middle Eastern	8	4.44			13	6.63		
South/Southeast Asian	41	22.78			38	19.39		
Multi-Ethnic/Other	22	12.22			13	6.63		
Sexual Orientation								
Asexual	4	1.77			11	5.61		
Bisexual	34	18.8			19	9.69		
Homosexual	15	7.20			5	2.55		
Heterosexual	113	61.3			150	76.53		
Prefer to self-identify	12	5.31			6	3.06		
Prefer not to disclose	2	0.88			5	2.55		

#### Judges of Online Dating Profiles

Judges between the ages of 18 to 30, living in Canada or the United States, single and seeking a relationship were recruited through Prolific, the participant pool at a Canadian university, social media (e.g., Facebook, Instagram), and the lab website. Participants who failed more than one attention check item in each survey, provided non-sensical responses to open-ended questions or acquiesced to Likert-type scale items measuring Big Five Traits were excluded. The final sample consisted of 196 judges (see Table 1). Participants received $20 CAD or 2 hr of course credit for completing all surveys.

### Measures and Procedures

#### Targets

Targets reported on individual difference measures (see OSF) and submitted screenshots of their online dating profiles.

##### Self-Perceptions and Desired Impressions

Targets reported on their self-perceptions of their personality and their desired impressions on Big Five Traits using the Ten Item Personality Inventory (TIPI; Gosling et al., 2003). All items were rated on a 1 = *strongly disagree* to 7 = *strongly agree* scale but prompts differed for self-perceptions (I think I am . . .) and desired impressions (I want people who see my profile to think I am . . .). See Table S1 for descriptive statistics.

##### Expressiveness

Expressiveness was measured as the sum of the number of photos (*M* = 3.46, *SD* = 2.34) and words (*M* = 29.16, *SD* = 40.92) on the profile, which were first *z*-scored across the entire sample and then summed.

##### Self-Esteem

Targets completed the Single Item Self-Esteem Scale (Robins et al., 2001), on a scale of 1 = *not very true of me* to 7 = *very true of me* (*M* = 4.79, *SD* = 1.55).

##### Relationship Goals

Targets responded to the question, “What kind of relationship are you seeking?.” This variable was collapsed into long-term relationship seeking (long-term monogamous or open relationship) and short-term relationship seeking (short-term monogamous, open, or casual sexual relationship). The number of targets seeking different types of relationships can be found in Table S2.

##### Attachment

Targets completed the 12-item Experience in Close Relationships Scale (Wei et al., 2007), which measures attachment anxiety (*M* = 3.50, *SD* = 0.79, α = .66) and avoidance (*M* = 3.24, *SD* = 0.69, α = .69) using items on a scale of 1 = *strongly disagree* to 5 = *strongly agree*.

##### Online Dating Profiles

Targets reported which online dating platforms they were currently active on and submitted screenshots of one of their online dating profiles. Profiles were mainly from Tinder, Bumble, and Hinge (see Table S3). Participants were required to submit a profile containing at least one photo, and remove identifying information beyond photos (e.g., names, location). The research team removed identifying information participants missed.

#### Judges

In survey 1, judges completed personality measures unrelated to this project (see OSF). Across three surveys administered on different days, judges rated 30 dating profiles (10 profiles in each survey) on the TIPI (Gosling et al., 2003) using a 1 to 7 scale (see Table S1 and Figure S1a).

##### Attractiveness

Judges rated each target on the item “I think this person is attractive/good-looking” on a scale of 1 = *strongly disagree* to 7 = *strongly agree* (*M* = 4.65, *SD* = 1.60).

#### Outcomes

##### Swiping

Judges indicated how they would respond to the target’s profile by moving a slider to the left or right in response the item “If you were to be matched with this person on an online dating platform, would you swipe left (No - 0) or right to accept them (Yes - 1)?.”

### Analyses

We used the Social Accuracy Model (SAM; Biesanz, 2010), which is a multilevel model that indexes profile agreement about characteristic patterns of traits (e.g., if Tom sees himself as being more outgoing than kind and more kind than hostile, does Julie also see him in this way?). In SAM, agreement on each item is modeled at level 1, while level 2 reflects an individual’s level of agreement across items. For all analyses, we used the psych, (Revelle, 2022), tidyverse (Wickham et al., 2019), car (Fox & Weisberg, 2019), moments (Komsta & Novomestky, 2015) and lmerTest (Kuznetsova et al., 2017) packages in R v.4.2.1 (R Core Team, 2022). We review core features of our models next, followed by specific models below.^3^

#### Decomposing Agreement

In all models we decompose raw agreement into a distinctive component (i.e., agreement about what makes a person distinctive) and a normative component (i.e., impressions that correspond with the average profile). To do so, we center the raw predictor profile (e.g., each person’s self-report) on the normative profile (i.e., the sample mean of each item of the main predictor profile) to create a distinctive profile for each person. We then predict the outcome from the grand mean-centered distinctive profile, which reflects the substantive aspects of personality, and the grand mean-centered normative profile, which reflects the normative and evaluatively positive aspects of personality (Rogers & Biesanz, 2015; Wood & Furr, 2016). The normative profile can be further decomposed into normativity and positivity,^4^ but, given the strong relationship between positivity and the normative profiles of self-perception (*r* = .94, 95% CI = [.76, .99], *p* < .001) and self-presentation (*r* = .99, 95% CI = [.94, 1.00], *p* < .001), we considered normativity to be redundant with positivity. As such, we refer to this normative profile as “*Positivity*” in all models going forward. Given that *Positivity* can reflect a statistical artifact (e.g., most people are more kind than cruel) and/or genuine agreement (e.g., Julie realizes that Tom is more kind than cruel) and is thus ambiguous, our main focus is on distinctive agreement.

The SAM also teases apart sources of accuracy, specifically the degree to which targets, judges, and dyads contribute to accuracy by modeling random slopes for each component. To quantify variance in agreement for targets, we report the *SD* of the random effects. Notably, past work on individual differences in who is a good target online range from *SD* = 0.09 on Twitter (Orehek & Human, 2017) to *SD* = 0.36 on Facebook (Human et al., 2021).

#### How Do People Want to Be Seen in Online Dating?

To test the extent to which targets *wanted* to be seen accurately and positively, *Positivity* was subtracted from each self-perception profile to create distinctive self-perception profiles *(Targets’ Self-Perceptions*), and both were entered as predictors of the target’s desired impression. *Targets’ Self-Perceptions* represents the extent to which each target wanted to be seen in line with their substantive and distinctive pattern of traits, and *Positivity* represents the extent to which targets wanted others to see their positive qualities, which may reflect reality, or simply the normative, socially desirable pattern of traits (Figure 1).

**Figure 1.**
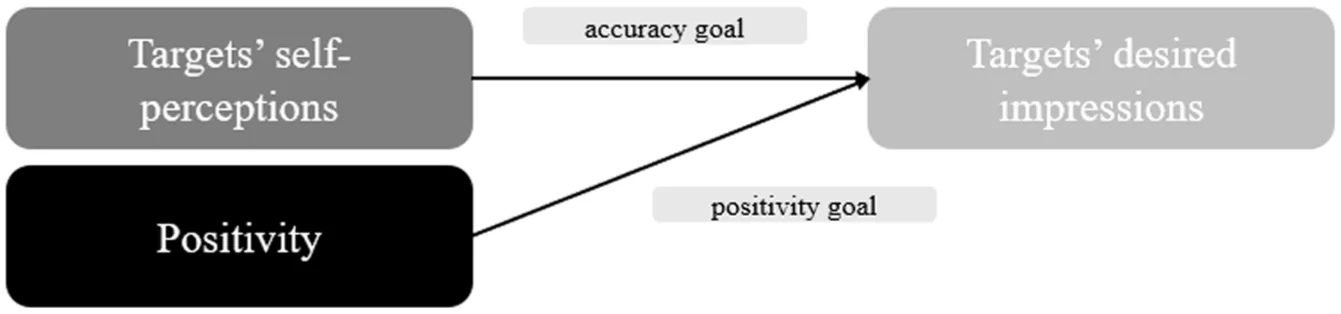
Self-Presentational Goals Model.

#### Are People Seen Accurately and/or in Line With Their Self-Presentational Goals?

##### Accuracy

To test whether target’s personalities were accurately perceived in online dating, we changed the dependent variable from desired impressions to how each target was seen by judges on each item. In this model (Figure 2A), our focus is on distinctive accuracy (represented by *Targets’ Self-Perceptions*) which represents the extent to which each target was seen in line with their substantive and distinctive pattern of traits.

**Figure 2.**
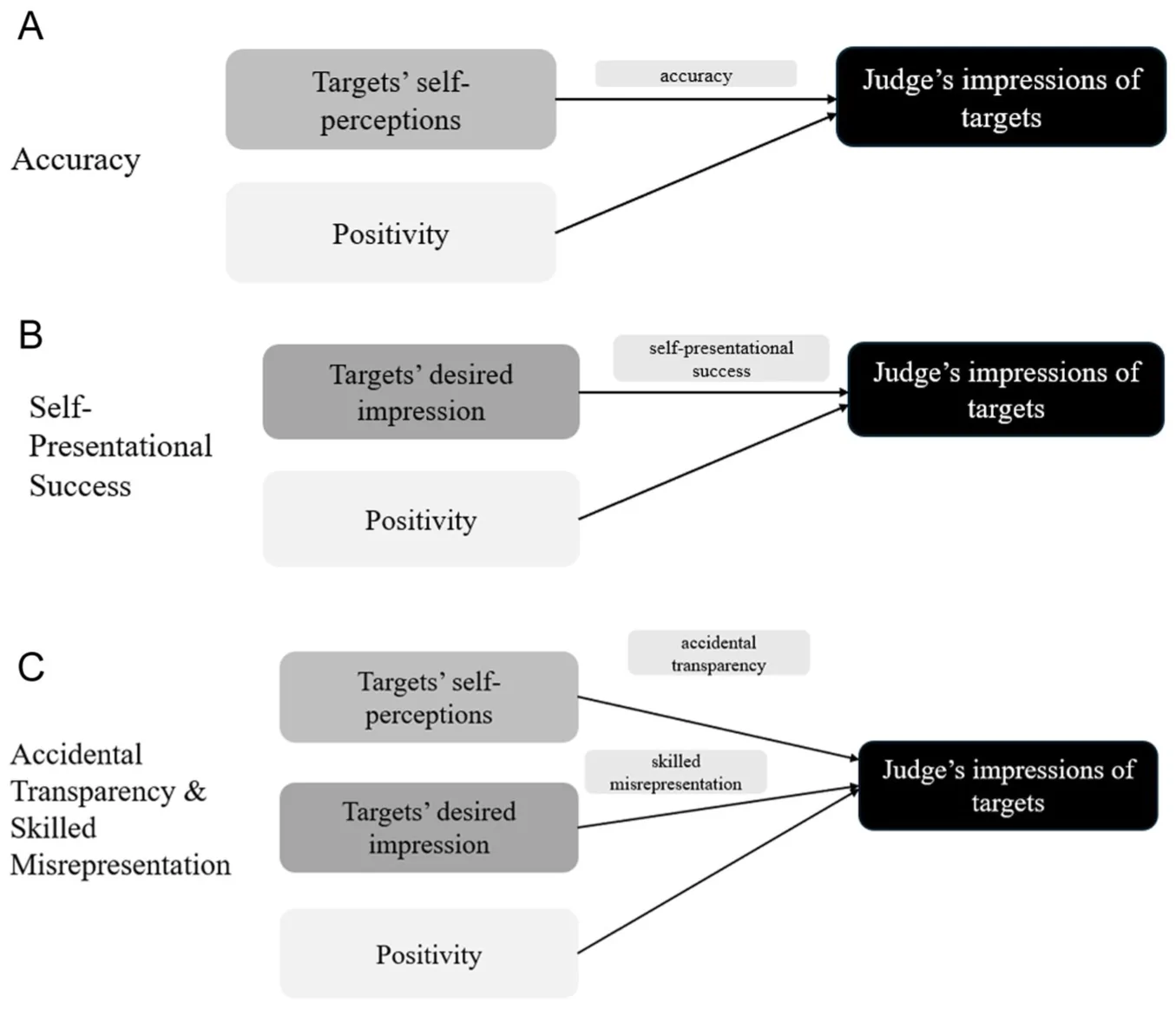
(A) Accuracy, (B) Self-Presentational Success, (C) Accidental Transparency, and Skilled Misrepresentation Models.

##### Self-Presentational Success

To test the degree to which people were seen as they wanted to be seen, we modified the accuracy model by replacing self-perception as a predictor with the self-presentational profile (*Targets’ Desired Impressions;* Figure 2B). Given this change, *Positivity* was the average desired impression rather than the average self-perception.^5^ Desired impressions reflect self-presentational success, or the extent to which each target was seen in line with their desired substantive and distinctive pattern of traits (regardless of whether this desired impression was accuracy or not).

##### Accidental Transparency and Skilled Misrepresentation

We included self-perception *(Targets’ Self-Perceptions)* and self-presentational goals (*Targets’ Desired Impressions*) as predictors of judge impressions (Figure 2C), which were both centered on the average self-perception of the sample. The self-perception coefficient reflects accidental transparency, or the degree to which a target’s personality leaked through despite wanting to be seen differently. The desired impression coefficient reflects skilled misrepresentation, or the degree to which a target was successful in presenting their personality differently from how they see themselves.

Taken together, these three models can provide a clearer picture about agreement in online dating. If we observe accuracy and self-presentational success, but we do not observe skilled misrepresentation, then much of why people were successful in self-presentation was because they wanted to be seen as they see themselves. Likewise, if we do not observe accidental transparency in the full model but do observe accuracy and self-presentational success, then people were transparent because they wanted to be.^6^

#### Outcomes and Moderators

To test outcomes (swiping) and moderators, we included swiping, expressiveness, self-esteem, attractiveness, relationship goals, and attachment as level 2 moderators in individual models. For swiping, we included both target and dyadic effects, which represents a target’s reputation for being picked and whether a particular judge chose to swipe yes on a particular target, respectively. For target effects, we calculated the proportion of “yes” swipes a target received, grand mean centered this value and included it as a moderator. For dyadic effects, swiping is a binary variable (judges either select 0 or 1), so we included each judge’s response to the item for each target as the moderator.

For moderators, the *z*-scored expressiveness score was entered directly into the model, and relationship goals was entered into the model as a dummy variable. Self-esteem, attachment anxiety, and attachment avoidance were grand mean-centered before being added as level 2 moderators in the models.

For attractiveness, we indexed attractiveness at the target level by exporting the Empirical Bayes estimates of judges’ ratings of target’s attractiveness for each target (i.e., their target effect), which represents how attractive each target was seen by the judges that rated them. Target variance was significant (*SD* = 0.64, *p* < .001), suggesting judges agreed on who was attractive. We entered this centered value directly into the model as a level 2 moderator.

Given that attraction can also be largely dyadic (Back et al., 2011; Joel et al., 2017), we included dyadic attractiveness (i.e., how attractive each judge uniquely saw each target) as a moderator. To do so, we exported the Empirical Bayes estimates of attractiveness ratings for each judge (i.e., perceiver effect), which represents how attractive each judge tends to see targets in general. From each judge’s raw attractiveness score of each target, we subtracted (a) the target effect, (b) the perceiver effect, and (c) the average attractiveness across all judges and targets to get how each judge rated each target accounting for how attractive targets were seen on average and how attractive judges tended to rate targets on average.

Effect sizes were calculated as a 2-standard deviation (*SD*) change in the moderator variable divided by the *SD* of the random effect estimate for the predictor in the base model (i.e., Gelman’s *d*; Gelman, 2008; Kerr et al., 2020). Given that Gelman’s *d* is comparable to Cohen’s *d* (Gelman, 2008), we assume *d* = .20 (*r* = .10) is a small effect, *d* = .41 (*r* = .20) is a medium effect, and *d* = .63 (*r* = .30) is a large effect (Funder & Ozer, 2019; Richard et al., 2003). A power analysis using GPower (Faul et al., 2007) for a point biserial correlation indicates that 180 targets would allow us to detect a medium effect, assuming α = .05 for a two-tailed test with .81 power.

## Results

### How Do People Want to Be Seen in Online Dating?

People varied significantly in the degree to which they wanted to be seen accurately, meaning that some people were more motivated to be seen as they saw themselves (Table 2 and Figure S2). Despite this variability, all targets demonstrated positive associations between their self- and desired impressions, suggesting no one wanted to be seen completely differently from their true personality.

**Table 2.** Fixed Effects and Random Effects for Goals, Accuracy, and Self-Presentational Success.

Model parameters	Fixed effect	Target random variance
	*b* (*SE*)	*SD*
Self-presentational goals model		
Accuracy goals	0.37*** (0.02)	0.20***
Positivity	1.42*** (0.03)	0.33***
Accuracy model		
Accuracy	0.04*** (0.01)	0.09***
Positivity	0.44*** (0.02)	0.25***
Self-presentational success model		
Self-presentational success	0.05*** (0.01)	0.11***
Positivity	0.30*** (0.01)	0.18***
Accidental transparency and skilled misrepresentation model		
Accidental transparency	0.01 (0.01)	0.08***
Skilled misrepresentation	0.07*** (0.01)	0.11***
Positivity	0.41*** (0.02)	0.24***

*Note*. Models including random variance for judges caused convergence issues along with tiny judge random variance; thus, we only model target random variance. Significant results are bolded.

*** *p* < .001. **p < .01. *p < .05.

### Are People Seen Accurately and/or in Line With Their Self-Presentational Goals?

We observed low but significant accuracy (Table 2), suggesting that targets’ personalities were observable to judges through their online dating profiles. However, like other online contexts (e.g., Twitter; Orehek & Human, 2017), the variability in how accurately targets were seen was small (*SD* = .09) and normally distributed (Figure S3a), suggesting it might be statistically difficult to identify moderators of accuracy. As a post hoc test, we explored if accuracy goals were associated with being seen accurately by exporting the Empirical Bayes estimates of the random slopes of accuracy goals in the Self-Presentational Goals model and accuracy in the Accuracy model. We found a small to medium positive association (*r* = .16, 95% CI = [.02, .30], *p* < .05), suggesting that targets who were seen more accurately tended to want to be seen that way (i.e., accuracy tends to be intentional).

Targets were also generally successful at conveying their desired impression (Table 2), regardless of whether their goal was to be seen accurately or not. Targets varied meaningfully in their success (*SD* = .11), in a normally distributed fashion (Figure S3b), but again this variance was small, suggesting it might be statistically difficult to identify moderators. We tested the association between accuracy and self-presentational success post hoc to confirm our assumption that part of self-presentational success reflects being seen accurately and found a large, positive association (*r* = .56, 95% CI = [.45 .66], *p* < .001), further suggesting that indeed, part of being seen accurately is intentional.

Were people successful in presenting themselves differently than their self-view? The answer seems to be yes given the significant effects for skilled misrepresentation and null effects for accidental transparency (i.e., personality did not accidentally leak out; Table 2). This suggests that self-presentational success is not *only* due to people wanting to be transparent; rather, people are also able to skillfully curate their desired impression without their true personalities leaking through.

### Is Accuracy and/or Self-Presentational Success Desirable?

Wanting to be seen accurately and generally being seen accurately by judges were not related to being chosen more (or less) by judges (swiping; Table 3). However, being seen accurately by a particular judge was negatively associated with being chosen by that judge, although this was a small to medium effect. Thus, it seems that judges are less interested in the targets they are more accurate about, suggesting that accuracy might have negative implications in online dating.

**Table 3.** Swiping Outcomes of Goals, Accuracy, and Self-Presentational Success.

Model parameters	Percentage of Yes swipes		Dyadic swiping	
	*b*	*d*	*b*	*d*
	(*SE*)	[95% CI]	(*SE*)	[95% CI]
Self-presentational goals model				
Accuracy goals	0.03 (0.12)	0.07 [−0.41, 0.55]	/	/
Accuracy model				
Accuracy	−0.05 (0.04)	−0.21 [−0.57, 0.15]	−0.03** **(0.01)**	−**0.28** **[−0.49, −0.08]**
Self-presentational success model				
Self-presentational success	−0.07 (0.06)	−0.26 [−0.65, 0.13]	−0.03* **(0.01)**	−**0.23** **[−0.43, −0.03]**
Accidental transparency and skilled misrepresentation model				
Accidental transparency	−0.04 (0.04)	−0.18 [−0.60, 0.25]	−0.03** **(0.01)**	−**0.38** **[−0.63, −0.13]**
Skilled misrepresentation	0.03 (0.05)	0.11 [−0.26, 0.47]	0.02 **(0.01)**	0.15 **[−0.01, 0.35]**

*Note*. We report effect size estimates, ds, for all interaction effects, calculated as a 2-standard deviation change in the moderator variable divided by the *SD* of the random effect estimate for the predictor in the base model (i.e., Gelman’s d). This estimate is comparable to Cohen’s *d* (see Gelman, 2008). Results for positivity can be found in Table S5. Significant results are bolded.

*** *p* < .001. **p < .01. *p < .05.

Self-presentational success was also not related to being chosen by judges in general but being seen in line with one’s goals by a specific judge was negatively associated with being chosen by that judge, although the effect was small. While small, this effect suggests that judges are less interested in the targets who successfully self-present to them.

Finally, neither accidental transparency nor skilled misrepresentation was associated with being chosen more overall. However, if a target’s true personality leaked out for a particular judge, that judge was less likely to swipe yes on the target. This medium effect size together with null effects for skilled misrepresentation suggests that judges might be less interested in targets whose personalities accidentally leak out to them, but there do not seem to be costs or benefits associated with skilled misrepresentation.

### Moderators of Accuracy and Self-Presentation

#### Expressiveness

In contrast to our predictions, targets who wanted to be seen more accurately did not include more information on their profile, and they were not seen more accurately (see Table 4 for moderator results). Instead, expressiveness predicted greater skilled misrepresentation. This medium effect suggests that targets who included more information on their profile were able to convey an impression that was different from their true personality.^7^

**Table 4.** Moderation of Expressiveness, Self-Esteem, Relationship Goals and Attractiveness With Goals, Accuracy, and Self-Presentational Success.

Model parameters	Expressiveness		Self-esteem		Relationship goals		Attractiveness		Dyadic attractiveness	
	*b*	*d*	*b*	*d*	*b*	*d*	*b*	*d*	*b*	*d*
	(*SE*)	[95% CI]	(*SE*)	[95% CI]	(*SE*)	[95% CI]	(*SE*)	[95% CI]	*(SE*)	[95% CI]
Self-presentational goals model										
Accuracy goals	−0.02 (0.01)	−0.02 [−0.49, 0.46]	0.06*** (0.01)	**0.98 [0.55, 1.40]**	0.09 (0.05)	0.41 [−0.07, 0.90]	0.00 (0.04)	0.00 [−0.47, 0.47]	/	/
Accuracy model										
Accuracy	0.00 (0.01)	0.15 [−0.22, 0.53]	0.00 (0.01)	0.10 [−0.23, 0.43]	0.02 (0.02)	0.25 [−0.22, 0.53]	−0.01 (0.01)	−0.12 [−0.49, 0.25]	−0.01* (0.00)	−**0.29 [**−**0.54**, −**0.04]**
Self-presentational success model										
Self-presentational success	—	—	−0.01 (0.01)	−0.22 [−0.60, 0.16]	−0.00 (0.02)	−0.01 [−0.42, 0.40]	−0.04 (0.02)	−0.40 [−0.83, 0.02]	−0.00 (0.00)	−0.10 [0.31, 0.10]
Accidental transparency and skilled misrepresentation model										
Accidental transparency	−0.00 (0.00)	−0.04 [−0.46, 0.38]	−0.00 (0.01)	0.00 [−0.44, 0.44]	0.03 (0.02)	0.30 [−0.11, 0.70]	0.00 (0.01)	0.02 [−0.43, 0.46]	−0.01*** **(0.00)**	−**0.54** **[−0.82, −0.26]**
Skilled misrepresentation	**0.02** * **(0.01)**	**0.50** **[0.13, 0.87]**	−0.00 (0.01)	−0.11 [−0.49, 0.27]	−0.01 (0.02)	−0.06 [−0.44, 0.32]	0.01 (0.02)	0.05 [−0.33, 0.44]	0.02*** **(0.00)**	**0.42** **[0.21, 0.62]**

*Note*. In the model testing expressiveness and self-presentational success, the random slope for self-presentational success was removed to resolve convergence errors. Results for positivity can be found in Table S6. Significant results are bolded.

*** *p* < .001. **p < .01. *p < .05.

#### Self-Esteem

In line with our predictions, targets higher in self-esteem wanted to be seen more accurately than others, a large effect, but self-esteem was not related to accuracy (or self-presentational success). Thus, despite wanting to be seen more accurately, targets higher in self-esteem were not seen any more accurately than others.

#### Relationship Goals

We predicted long-term relationship seekers would want to be seen more accurately but we did not observe this effect. Furthermore, in contrast to predictions, the type of relationship a target was seeking was not related to accuracy (or self-presentational success).

#### Attractiveness

Being rated as more attractive overall was not related to wanting to be seen accurately, accuracy, or self-presentational success (or accidental transparency). Dyadic attractiveness was negatively associated with accuracy, though this was a small effect to medium effect, and was unrelated to self-presentational success. However, it was related to less accidental transparency and more skilled misrepresentation, both displaying medium effects. That is, targets who were seen as more attractive by particular judges made their desired impression on those judges, without their true personalities leaking out.

#### Attachment

In line with our predictions, both anxiously and avoidantly attached individuals wanted to be seen less accurately than more securely attached individuals, although the effect was large for avoidantly attached individuals and medium to large for anxiously attached individuals (Table 5). However, despite these goals, neither anxious nor avoidant attachment was related to accidental transparency or skilled misrepresentation.

**Table 5.** Moderation of Attachment With Goals, Accuracy, and Self-Presentational Success.

Model parameters	Anxious attachment		Avoidant attachment	
	*b*	*d*	*b*	*d*
	(*SE*)	[95% CI]	(*SE*)	[95% CI]
Self-presentational goals model				
Accuracy goals	−0.07* (0.03)	**−0.54 [−1.00, −0.07]**	−0.11**(0.03)	**−0.77 [−1.23, −0.31]**
Accuracy model				
Accuracy	0.01 (0.01)	0.17 [−0.16, 0.50]	−0.01 (0.01)	−0.16 [−0.47, 0.15]
Self-presentational success model				
Self-presentational success	0.01 (0.02)	0.19 [−0.21, 0.60]	0.02 (0.02)	0.19 [−0.24, 0.62]
Accidental transparency and skilled misrepresentation model				
Accidental transparency	0.02 (0.01)	0.32 [−0.12, 0.77]	−0.01 (0.01)	−0.18 [−0.63, 0.27]
Skilled misrepresentation	−0.01 (0.01)	−0.08 [−0.46, 0.30]	0.02 (0.02)	0.23 [−0.17, 0.64]

*Note*. In the model testing expressiveness and self-presentational success, the random slope for self-presentational success was removed to resolve convergence errors. Results for positivity can be found in Table S7. Significant results are bolded.

*** *p* < .001. **p < .01. *p < .05.

## Discussion

How do online daters want to be seen online and are judges able to see daters clearly? Overall, online daters were seen accurately (mostly because they want to be) but were also able to successfully self-present an image of themselves that was different from their general self-image. Thus, when people have the opportunity to curate cues, they present themselves authentically (Back et al., 2010), but perhaps given the high-stakes context, they also try (and do) project a different image. Importantly though, being authentic (even when intentional) did not seem to pay off– being seen accurately was related to being liked less. Next, we explore the deeper implications of these effects for romantic attraction and interpersonal perception.

### Accuracy and Accidental Transparency

We tested the degree to which people’s personalities are seen accurately via their online dating profiles, both when they want to be seen accurately and when they do not. To our knowledge, this is the first line of work that has made a distinction between accuracy that is intentional versus unintentional, a distinction that is critical when people are motivated to self-present and when they can carefully curate a desired impression (e.g., in an online context). People varied in the degree to which they wanted to be seen accurately and overall, targets were seen accurately when they wanted to be. Our results suggest that accuracy was mostly intentional, which adds to the growing body of work suggesting that targets largely control accuracy (Human & Biesanz, 2013). Indeed, theory (Funder, 1995) and empirical evidence (Rogers & Biesanz, 2019) suggest that accuracy is largely due to targets being transparent rather than due to judges being keen observers of cues. Building on this, the current work suggests that transparency might also be intentional—targets want to be seen transparently and are choosing to convey more, valid cues. Future work applying our accuracy versus accidental transparency approach might identify other contexts where accuracy is intentional versus accidental. Perhaps contexts where targets cannot curate cues as easily (e.g., speed dating) might show more accidental transparency.

#### Is Accuracy Desirable?

Targets who were seen more accurately overall did not receive more “yes” swipes from judges overall, but targets who were seen more accurately by particular judges (accidental or not) were chosen less by those judges. These dyadic effects resemble findings from speed dating (Kerr et al., 2020), suggesting that the associations between romantic interest and accuracy may occur at the dyadic level rather than the target level. This may be due to weak target effects for accuracy (i.e., people are not substantially and reliably more accurate about some targets than others) but also due to the idiosyncratic nature of swiping (i.e., attraction is more dyadic than general; Back et al., 2011; Luo & Zhang, 2009).

Why might accuracy be related to lower romantic interest? Previous work in speed dating has suggested that more transparent targets might be revealing their personalities in ways that are inconsistent with dating norms, thereby reducing romantic interest (e.g., excessive self-disclosure; Kerr et al., 2020). However, as all judges see the same profile for a given target, if this were the case, we would expect to see a general effect, which we did not. Thus, it is likely that this effect is due to how each judge uniquely processes a target’s cues in ways that result in judges paying less attention to cues when they are attracted to a person. One possibility could be that halo biases result in judges perceiving targets they find more physically attractive less accurately (Anusic et al., 2009). However, a post hoc test demonstrated that physical attractiveness did not explain the link between romantic interest and accuracy (Table S11), adding to previous work that has also found mixed results for this link (Kerr et al., 2020). Future work might explicitly test how romantic interest is related to lower accuracy, perhaps by exploring judges’ unique relational history (Zayas & Shoda, 2007) or the role of assumed similarity in liking (Tidwell et al., 2013). Regardless, this finding suggests that stated preferences for authenticity might be misguided (Wotipka & High, 2016), which adds to a growing body of work suggesting daters stated preferences do not always match actual preferences (Eastwick & Finkel, 2008).

#### Moderators of Accuracy Goals and Being Seen Accurately

We explored who *wanted* to be seen more accurately and who *was* seen more accurately than others in online dating. In line with our predictions, individuals high in self-esteem wanted to be seen more accurately, while anxiously and avoidantly attached targets wanted to be seen less accurately. However, in contrast to predictions, their motivations for accuracy did not translate into actual accuracy. Indeed, while the individual differences we explored have been associated with accuracy in past work, none of our individual differences predicted overall accuracy, suggesting a potential boundary condition to the cross-contextual consistency of being a good target (Human et al., 2021; Wallace & Biesanz, 2021).

One reason for these null effects might be that accuracy and accidental transparency did not vary enough systematically across targets to identify who is seen more accurately. Perhaps when there is the opportunity to meet a target, judges are more motivated to differentiate among targets, which in turn might boost overall levels of and variance in accuracy. Alternatively, it is possible that online dating apps simply offer limited ways for judges to detect and utilize cues.

### Successful Self-Presentation and Skilled Misrepresentation

Another goal of this study was to test if people can successfully curate cues online to craft the impressions they want to make. We found that people were successful at conveying their desired impressions, even when they wanted to be seen differently than how they see themselves (skilled misrepresentation), suggesting that people can curate cues successfully online.

While self-presentational goals and success were related to accuracy (i.e., people tended to want to be seen accurately and were), an important part of self-presentational success was about misrepresenting the self. What exactly did people want to misrepresent? On average, people wanted to seem more emotionally stable (Table S1), but individuals varied in the ways they wanted to self-present differently than how they saw themselves (Figure S1b). Future work might explore individual differences in self-presentation for specific traits to better understand who wishes to misrepresent different traits and/or which traits they are generally more successful at misrepresenting.

Taken together with the small effects of accuracy and null effects for accidental transparency, these findings have implications for online daters who hold the subjective idea that they are screening for valid information online (Ellison et al., 2006). Indeed, whether they are viewing the profiles of daters who are motivated to show or hide their true selves, they might be missing key cues. Notably, this might contribute to some of the frustration online daters experience if they are not able to accurately judge the personalities of the people they meet, leading to disappointment during in-person meetings.

#### Is Self-Presentational Success Desirable?

Being able to curate a particular impression could be a valuable skill in online dating if it helped daters foster more romantic interest from judges. However, we found negative associations between swiping and self-presentational success (although not with skilled misrepresentation), suggesting that when daters convey their desired impression, they are liked less. What went wrong? Given that part of self-presentational success includes being seen accurately, it is likely that the negative associations between romantic interest and accuracy are driving these findings. Notably, even when targets were successful at conveying an impression that is different from their personality (skilled misrepresentation), they were not especially liked. Therefore, rather than judges detecting and appreciating a socially skilled dater, it seems as though targets’ desires to be seen accurately might backfire.

#### Moderators of Self-Presentation

We expected that both anxiously and avoidantly attached individuals would want to be seen differently from their true personality but only avoidantly attached individuals would be successful. However, we did not find any evidence that attachment was related to skilled misrepresentation. Instead, we found that people who included more information on their profiles were more skilled at misrepresentation (rather than seen more accurately as we expected), further highlighting targets’ ability to curate cues for a desired impression. Future work should investigate which cues daters use to be seen in particular ways (e.g., Tom includes pictures with friends and lots of text to seem more social), and which cues are successful (e.g., group pictures influence perceptions of sociability but lots of text does not).

Beyond target’s motivations, we observed other factors related to how targets were seen. Specifically, targets who were seen as more attractive by particular judges were able to skillfully misrepresent their personality to those judges. Past work suggests that attractiveness can foster accuracy by promoting attention toward targets (Lorenzo et al., 2010; Tissera et al., 2023). However, if some targets are curating cues to misrepresent their personality, increased attention might foster skilled misrepresentation, as we see here. Future work should investigate other key moderators related to having a strong motivation to self-present (e.g., Dark Triad traits; Willis et al., 2023) or the ability to self-present (e.g., experience with online dating).

### Limitations and Future Directions

The current research is the first, to our knowledge, to explore accuracy and self-presentation in online dating, as well as consequences of and individual differences in these processes. While the study was high powered and used ecologically valid profiles, there are several limitations that future research might address.

First, the online dating process can be considered high stakes when targets and judges expect to meet in person. However, judges in our study did not expect to meet targets, potentially lowering motivation to see targets accurately (Biesanz & Human, 2010). Therefore, future work should test these processes when judges can meet targets to better capture the high-stakes nature of dating. Relatedly, future work should explore accuracy and self-presentation as people move from online to in person. While accuracy seems to be consistent across some contexts (Human et al., 2021), it is possible that self-presentation is context-specific, such that the people who successfully curate cues online do not necessarily make their desired impressions in person. For example, the expressive target might be a successful self-presenter online if they know how to appropriately curate cues, but accidentally transparent in person if their expressive nature allows more of their personality to leak out. Overall then, studying these processes when people can (and do) meet in person might reveal boundary conditions in terms of how they unfold and who is successful in dating.

While we focused mainly on the target, individual differences in judges might also be critical to study both in the online and in person aspects of dating (Letzring, 2008; Rogers & Biesanz, 2019). Indeed, a judge’s own social and relationship history can affect how they attend to cues in potential romantic partners (Zayas & Shoda, 2007), suggesting judges may play an important role in accuracy. While there may be little variability in judge accuracy when passively viewing profiles, judges can likely extract unique cues via chatting functions or in person (e.g., Julie’s warm disposition encourages Tom to disclose more, thereby providing more personality cues; Letzring, 2008). Thus, a good judge might emerge at later phases of dating. In addition, there may be a good judge of self-presentation (i.e., someone who can detect misrepresentation more than others), which was not a focus of our study, but should be explored in future work.

In addition, while we investigated *who* is seen more accurately than others, future work could investigate *which traits* are seen more accurately, to determine if there are parts of people’s personalities that are reliably seen by others versus those that leak out. Importantly, there could be traits beyond the Big Five that are especially important in dating (e.g., funny, confident) that targets are motivated to misrepresent or that judges are motivated to detect accurately. Future work should explore these processes using a trait-by-trait approach.

Finally, our work used targets’ general self-perceptions as an accuracy criterion, but future work could explore what targets consider to be an accurate self-portrayal in dating specifically. For example, some targets might have a self-view that is specific to the dating context (e.g., Tom is very conscientious and somewhat agreeable usually, but in dating, he is highly agreeable). Future work can explore how people see their personality in a dating context, and how this is related to the impressions they try to convey in early dating.

#### Constraints on Generalizability

While targets and judges were ethnically diverse, all participants were between the ages of 18 and 30 and residing in Canada or the United States, many of whom were enrolled in university. As such, this research can mainly be generalized to young adults in online dating. While younger adults might be the most active in online dating, older adults might have different self-presentational goals for dating. Future research should extend this research to older adults and beyond North America.

## Conclusion

Overall, this study sheds light on the self-presentation strategies online daters use, the success of these strategies, and who uses these strategies. Online daters tend to want to be both transparent and misrepresent themselves, and they are somewhat successful in both goals. However, transparency seems to backfire when it comes to romantic attraction, despite the intuition that being real fosters better dating experiences. With respect to who uses or is successful with an accuracy or misrepresentation strategy, we were only able to identify factors that hinder these strategies (e.g., attractiveness). Thus, future work is needed to identify factors that promote these strategies. Finally, this work also advances interpersonal perception research by demonstrating the important role of the target’s motivation to be seen accurately and the need to make the distinction between intentional and accidentally transparency more generally in interpersonal perception research in other contexts. Interpersonal perception work should continue to explore targets’ goals when studying accuracy to better understand the interconnected goals (and outcomes) of transparency versus misrepresentation.

## Supplemental Material

sj-docx-1-psp-10.1177_01461672251386488 – Supplemental material for Who Did I Swipe On? Accuracy and Self-Presentation in Online Dating Supplemental material, sj-docx-1-psp-10.1177_01461672251386488 for Who Did I Swipe On? Accuracy and Self-Presentation in Online Dating by Sarra Jiwa, Norhan Elsaadawy and Erika N. Carlson in Personality and Social Psychology Bulletin

## Data Availability

All data relevant to the research are available within the article.

## Notes

1. Notably, all results for swiping decisions replicated when using Likert-type liking ratings (see Table S9).

2. Details regarding the recruitment process and procedures used for screening and excluding ineligible participants are included in Supplemental Materials, page 3.

3. Equations can be found in the Supplemental Materials on pages 10 to 12.

4. See the Supplemental Materials for an explanation of how the positivity profile was indexed and how the normative profile was decomposed into normative and positive components (page 13). Table S8 shows results of the models that included normativity and positivity as separate predictors.

5. The average self-perception and average desired impression were strongly correlated (*r* = .97, 95% CI = [.86, .99], *p* < .001). As other models consider positivity as the average self-perception, results of the model with the average self-perception as positivity are in the SOM (page 23).

6. Given that we are primarily interested in targets, we do not model judge variance in these models. See Table S10 for results including judge variance.

7. Given that different platforms (e.g., Hinge, Tinder) allow users to include different amounts of information, and the number of photos and words differed based on the online dating platform (Table S3), we tested if the platform type explained the effects of expressiveness. We did not find any effects of platform type across all models, nor did platform type explain the link between expressiveness and skilled misrepresentation.
