# Supplementary material for: Who Did I Swipe On? Accuracy and Self-Presentation in Online Dating
Source: Pers Soc Psychol Bull. 2025 Nov 13;52(11):3720–35. doi: 10.1177/01461672251386488 (PMC13646434; doi:10.1177/01461672251386488)
Supplement: Supplementary file 1 [file sj-docx-1-psp-10.1177_01461672251386488.docx]

**Supplemental Materials**

**Table of Contents**

**Tables**

[**Table S1**	5](#_Toc205900675)

[**Table S2**	6](#_Toc205900676)

[**Table S3**	7](#_Toc205900677)

[**Table S4**	9](#_Toc205900678)

[**Table S5**	15](#_Toc205900681)

[**Table S6**	16](#_Toc205900682)

[**Table S7**	18](#_Toc205900683)

[**Table S8**	19](#_Toc205900684)

[**Table S9**	21](#_Toc205900686)

[**Table S10**	22](#_Toc205900687)

[**Table S11**	23](#_Toc205900688)

**Figures**

[**Figure S1**	25](#_Toc205900690)

[**Figure S2**	26](#_Toc205900691)

[**Figure S3**	27](#_Toc205900693)

**Extra Notes**

[**Sample Size**	3](#_Toc194570504)

[**Sample Data and Equations for All Models**	9](#_Toc205292174)

[**Does the Normative Profile Adequately Reflect Positivity?**	13](#_Toc205292175)

[**Additional Moderators: Gender and Trait Observability**	19](#_Toc205292179)

[**Were Targets (Still) Seen in Line With Their Self-Presentational Goals in Alternate Models?**	23](#_Toc205292183)

# **Sample Size**

For both targets and judges, instead of solely recruiting from verified platforms (e.g., Prolific) or via the undergrad pool, we opened the study to a public domain, which invited opportunities for people to try to complete the study in disingenuous ways (i.e., bots). We used stringent quality controls (e.g., attention checks, scoring acquiesced on Big Five items within the same domain, requiring photo ID for questionable participants, to remove these disingenuous actors, explaining why the final sample size is much lower than the original number of participants included for both targets and judges. The specifics for how we excluded participants in each sample is described below.

***Targets***

We received a total of 765 submissions for targets. Targets were required to meet all of the eligibility criteria (they had to be between the ages of 18-30, living in Canada or the USA, single and seeking a relationship and be active on an online dating profile). Participants were required to upload a real online dating profile, pass all the attention check items, and provide sensical responses to all open-ended questions to be included. We screened participants for the above criteria as they completed the survey and aimed to collect data until we reached 200 targets that met all of the criteria, as per our pre-registered plan. We terminated data collection after we had 208 targets that completed the survey successfully. We then excluded participants who acquiesced to Likert scale items of Big Five Traits (i.e., responding with high values to both positively and negatively keyed items of the same trait). Specifically, for Big Five items measured on the Ten Item Personality Inventory, we took the average of two items measuring the same trait (one positively and one negatively keyed) and dropped participants that were greater than 1 SD away from the mean. The final sample consisted of 180 targets aged 18-30 years old (see Table 1 for demographics). Participants were compensated $15 CAD (or the USD equivalent).

***Judges of Online Dating Profiles***

We recruited judges between the ages of 18-30, living in Canada or the USA, single and seeking a relationship were recruited through Prolific, the participant pool at a Canadian university, social media (e.g., Facebook, Instagram), and the lab website. We had a total of 1294 submissions to the initial intake survey. Through social media advertising we received many duplicate submissions from people trying to complete the study multiple times under fake names or email addresses. Therefore, we used an extensive vetting process to catch these participants including verifying their locations, IP addresses, screening out participants that tried to complete the study multiple times if they failed the qualifying questions and ensuring participants did not fail more than one attention check. We also excluded participants who provided non-sensical responses to open-ended questions or acquiesced to Likert scale items using the same procedure used for targets. The final sample consisted of 196 judges (see Table 1 for demographics). Participants received $20 CAD (or the USD equivalent) for completing four 30-minute surveys. Participants recruited from the university pool were able to receive up to 2 hours of course credit for completing all four surveys.

# **Table S1**

*Descriptive Statistics of Targets’ Self-perceptions, Desired Impressions and Judges’ Impressions of Targets*

|  | Target ratings | | Judge ratings | |  |
| --- | --- | --- | --- | --- | --- |
| TIPI item | Self-Perception  *M* (*SD*) | Desired Impression  *M* (*SD*) | | Impressions  *M* (*SD*) | |
| Extraverted, enthusiastic | 4.75 (1.63) | 5.32 (1.35) | | 4.72 (1.53) | |
| Critical, quarrelsome | 3.51 (1.72) | 2.44 (1.69) | | 3.64 (1.47) | |
| Dependable, self-disciplined | 5.47 (1.31) | 5.44 (1.18) | | 4.51 (1.37) | |
| Anxious, easily upset | 3.67 (1.64) | 1.91 (1.07) | | 3.64 (1.39) | |
| Open to new experiences, complex | 5.75 (1.15) | 5.92 (1.03) | | 4.83 (1.37) | |
| Reserved, quiet | 4.07 (1.71) | 3.27 (1.42) | | 3.94 (1.56) | |
| Sympathetic, warm | 5.61 (1.24) | 5.77 (1.06) | | 4.53 (1.32) | |
| Disorganized, careless | 2.73 (1.51) | 1.87 (1.08) | | 3.47 (1.44) | |
| Calm, emotionally stable | 5.05 (1.54) | 5.52 (1.12) | | 4.39 (1.30) | |
| Conventional, uncreative | 2.54 (1.32) | 2.13 (1.29) | | 3.47 (1.46) | |

*Note*. TIPI items were rated on a 1-7 scale. Target self-perceptions, desired impressions and judge impressions are displayed in the Supplemental Materials (Figure S1)

# **Table S2**

*Number of Targets Seeking Different Types of Relationships*

|  | Long-term monogamous relationship | Long-term open relationship | Short-term monogamous relationship | Casual sexual relationship | Short-term open relationship |
| --- | --- | --- | --- | --- | --- |
| Number of Targets | 103 | 10 | 30 | 19 | 6 |
|  | Long-term total | | Short-term total | | |
|  | 113 | | 55 | | |

# **Table S3**

*Number of Target Dating Profiles Per Online Dating Platform*

|  |  | Type of Online Dating Platform | | | | | | | |
| --- | --- | --- | --- | --- | --- | --- | --- | --- | --- |
|  | Tinder | Bumble | OkCupid | Match.com | Facebook Dating | eHarmony | Hinge | Plenty of Fish | Other |
| Number of Targets | 87 | 26 | 10 | 0 | 1 | 2 | 41 | 0 | 13 |
| Mean Number of Photos | 3.14 | 3.27 | 2.10 | 0 | 1.00 | 2.00 | 5.10 | 0 | 2.23 |
| Mean Number of Words | 20.52 | 37.15 | 81.20 | 0 | 0 | 82.00 | 30.51 | 0 | 20.77 |

*Note.* Targets submitted online dating profiles from any platform they were currently active on. The number of targets that submitted a profile from each platform is shown in Table S3. Different online dating applications are known by users to facilitate different types of relationships, and as a result, have their own unique cultures and sets of norms which may influence the way daters behave on these platforms. For example, Tinder is primarily known as a “hookup” app, for people interested in casual, sexual relationships (Sevi, 2017), whereas Hinge advertises itself as a platform to help foster long-term relationships (Hinge, 2023). As a result, each of these applications allows users to include different amounts of information on their profile, and each allows a different level of specificity (e.g., Hinge allows users to specify the highest level of education a user has, whereas Tinder does not). Due to different cues that could be available on each of the apps, which is dictated by the platform itself, we tested for the effects of platform type on accuracy. Given the large number of apps that dating profiles were taken from, we collapsed the profile platform variable into three categories representing the most common applications in the study: Tinder, Hinge and Bumble, and added an “Other” category representing profiles from any other platforms. We did not find any effects of the profile platform on how targets wanted to be seen, or on accuracy, self-presentational success, accidental transparency, or skilled misrepresentation.

# **Table S4**

# *Sample Data and Equations for All Models*

| Target | Item | Desired Impression | Self- Rating | Positivity Desired Impression | Positivity Self-Rating | Distinctive Desired Impression  (centered on Positivity Desired Impression) | Distinctive Self Rating | Distinctive Desired Impression  (centered on Positivity Self-Rating) | Judges’ Average Rating of Tom |
| --- | --- | --- | --- | --- | --- | --- | --- | --- | --- |
| Tom | Extraverted, enthusiastic | 6 | 4 | 5.5 | 4.5 | 0.5 | -0.5 | 1.5 | 5 |
|  | Critical, quarrelsome | 3 | 4 | 1.5 | 2.5 | 1.5 | 1.5 | 0.5 | 2 |
|  | … TIPI Items |  |  |  |  |  |  |  |  |
| Tammy | Extraverted, enthusiastic | 7 | 5 | 5.5 | 4.5 | 1.5 | 0.5 | 2.5 | 6 |
|  | Critical, quarrelsome | 1 | 3 | 1.5 | 2.5 | -0.5 | 0.5 | -1.5 | 2 |
|  | …TIPI items |  |  |  |  |  |  |  |  |

***How do people want to be seen in online dating?***

Sample data for each of the following models can be found in Table S4.

To test the extent to which targets *wanted* to be seen accurately and positively*,* Positivity was subtracted from each self-perception profile to create distinctive self-perception profiles *(*TSelf), and both were entered as predictors of the target’s desired impression (Ytk) (Equation 1). Thus, distinctive self-perception 𝛽_1_*_t_* represents the extent to which each target wanted to be seen in line with their substantive and distinctive pattern of traits, and 𝛽_2_*_t_* represents positivity. The random variance around these values (*SD*) quantifies how much variance there is in targets who want to be seen accurately (*u*_1t_) or positively (*u*_2t_).

(1.1) *Y_tk_* = 𝛽_0_*_t_* + 𝛽_1t_(TSelf)*_k_* + 𝛽_2_*_t_*(Positivity)*_k_* + ɛ*_tk_*
(1.2) 𝛽_0_*_t_* = 𝛽_00_ + 𝛽_01_(Moderator)_t_ + *u*_0_*_t_*

𝛽_1_*_t_* = 𝛽_10_ + 𝛽_11_(Moderator)_t_ + *u*_1_*_t_*

𝛽_2_*_t_* = 𝛽_20_ + 𝛽_21_(Moderator)_t_ + *u*_2_*_t_*

***Ytk*** *= Desired Impression*

***TSelf*** *= Distinctive Self Rating*

***Positivity*** *= Positivity Self Rating*

***Are Targets Seen Accurately and/or in Line With Their Self-Presentational Goals?***

**Accuracy.** To test whether target’s personalities were accurately perceived in online dating, we modified Equation 1.1 and 1.2 by changing the dependent variable from desired impressions to how each target *t* was seen on average by judges on item *k*. In this model, our focus is on distinctive accuracy, or 𝛽_1_*_t_*, which represents the extent to which each target was seen in line with their substantive and distinctive pattern of traits. We also report on 𝛽_2_*_t_*_,_ the degree to which each target was seen positively, as well as the random variance around these values (*SD*), specifically *u*_1_*_t_* and *u*_2_*_t_*.

(Modified 1.1)	Y_tk_ = 𝛽_0t_ + 𝛽_1t_(TSelf)k + 𝛽_2t_(Positivity)_k_ + ɛ_tk_

(Modified 1.2)	𝛽_0t_ = 𝛽_00_ + 𝛽_01_(Moderator)_t_ + u_0t_

𝛽_1t_ = 𝛽_10_ + 𝛽_11_(Moderator)_t_ +  u_1t_

𝛽_2t_ = 𝛽_20_ + 𝛽_21_(Moderator)t +  u_2t_

***Ytk*** *= Judges’ Average Rating of Target*

***TSelf*** *= Distinctive Self Rating*

***Positivity*** *= Positivity Self Rating*

**Self-Presentational Success*.*** To test the degree to which people were seen as they wanted to be seen, regardless of if they wanted to be seen accurately, we modified the accuracy model by replacing self-perception as a predictor with the self-presentational profile (SelfPresent*)* (Equation 2). Given this change, positivity was the average desired impression rather than the average self-perception.^[1]^ In this model, we are primarily interested in self-presentational success, 𝛽_1_*_t_*, which represents the extent to which each target was seen in line with their desired substantive and distinctive pattern of traits (regardless of whether this desired impression was accuracy or not). We also report on positivity, 𝛽_2_*_t_*, which represents the degree to which each target was seen positively, and the random variance around these values (*SD*), *u*_1_*_t_* and *u*_2_*_t_*.

(2.1) *Y_tk_* = 𝛽_0_*_t_* + 𝛽_1_*_t_*(SelfPresent)*_k_* + 𝛽_2_*_t_*(Positivity)*_k_* + ɛ*_tk_*
(2.2) 𝛽_0_*_t_* = 𝛽_00_ + 𝛽_01_(Moderator)*_t_* + *u*_0_*_t_*

𝛽_1_*_t_* = 𝛽_10_ + 𝛽_11_(Moderator)*_t_* + *u*_1_*_t_*

𝛽_2_*_t_* = 𝛽_20_ + 𝛽_21_(Moderator)*_t_* + *u*_2_*_t_*

***Ytk*** *= Judges’ Average Rating of Target*

***SelfPresent*** *= Distinctive Desired Impression*

***Positivity*** *= Positivity Desired Impression*

**Accidental Transparency and Skilled Misrepresentation.** In our final model, we combined the accuracy and self-presentation models by including both self-perception *(*TSelf*)* and self-presentational goals (SelfPresent) as predictors, which were both centered on Positivity (the average self-perception) (Equation 3). In this model, we focus on accidental transparency, 𝛽_1_*_t_* , the degree to which a target’s personality leaked through despite wanting to be seen differently, and skilled misrepresentation, 𝛽_2_*_t_*, the degree to which a target was successful in presenting their personality differently from how they see themselves. Positivity, 𝛽_3_*_t_*, represents the degree to which targets were seen positively. The random variance around these values (*SD*) quantifies the variance in accidental transparency (*u*_1_*_t_*), skilled misrepresentation (*u*_2_*_t_*), and positivity (*u*_3_*_t_*).

(3.1) *Y_tk_* = 𝛽_0_*_t_* + 𝛽_1_*_t_*(TSelf)*_k_* + 𝛽_2_*_t_*(SelfPresent)*_k_* + 𝛽_3_*_t_*(Positivity)*_k_* + ɛ*_tk_*
(3.2) 𝛽_0_*_t_* = 𝛽_00_ + 𝛽_01_(Moderator)*_t_* + *u*_0_*_t_*

𝛽_1_*_t_* = 𝛽_10_ + 𝛽_11_(Moderator)*_t_* + *u*_1_*_t_*

𝛽_2_*_t_* = 𝛽_20_ + 𝛽_21_(Moderator)*_t_* + *u*_2_*_t_*

𝛽_3_*_t_* = 𝛽_30_ + 𝛽_31_(Moderator)*_t_* + *u*_3_*_t_*

***Ytk*** *= Judges’ Average Rating of Target*

***TSelf*** *= Distinctive Self Perception*

***SelfPresent*** *= Distinctive Desired Impression centered on Self*

***Positivity*** *= Positivity Self Perception*

Taken together, these three models can provide a clearer picture about agreement in online dating. If we observe accuracy and self-presentational success, but we do not observe skilled misrepresentation, then much of why people were successful in self-presentation was because they wanted to be seen as they see themselves. Likewise, if we do not observe accidental transparency in the full model but do observe accuracy and self-presentational success, then people were transparent because they wanted to be. ^[2]^

# **Does the Normative Profile Adequately Reflect Positivity?**

The normative component of agreement is a mix of normative information and positivity (i.e., what is normative is good; Rogers & Biesanz, 2015; Wood & Furr, 2016), so the normative profile can be further decomposed into normativity and positivity components. To do this, we computed a socially desirable profile, which is the mean social desirability ratings of each of the TIPI items from 54 coders. Some of these ratings were obtained for an unrelated study and as such were rated on a 1(very undesirable) to 15 (very desirable) scale in response to the question: “How socially desirable, or positive, is this trait?”, but we transformed these ratings to be on a 1-7 scale. The raters demonstrated very high agreement, (*ICC* (2,k) = 1.00, 95% CI [.99, 1.00], *F*(9, 468) = 241, *p* < .001. We centered the normative profile on this socially desirable profile, such that any overlap between the two is reflected in the socially desirable profile and included both profiles as predictors in all models. Therefore, we include the distinctive, normative and socially desirable profiles as predictors. The remainder of the normative component represents a statistical artifact and what is normative beyond what is socially desirable, although these are typically highly correlated. All profiles were grand mean centered before being entered into the models. Results of these full models are shown in Table S8. Notably, these results strongly mirror the simpler model from the main text, further confirming that using the simpler model with normativity as a proxy for positivity, is appropriate

# **Table S5**

| Model Parameters | Percentage of Yes Swipes | | | Dyadic Swiping | | |
| --- | --- | --- | --- | --- | --- | --- |
|  | *b* | | *d* | *b* | *d* | |
|  | (*SE*) | | [95% CI] | (*SE*) | [95% CI] | |
| Self-Presentational Goals Model | | | | | | |
| Accuracy | | 0.03  (0.12) | 0.07  [-0.41, 0.55] | / | / | |
| Positivity | | -0.15  (0.16) | -0.19  [-0.60, 0.22] | / | / | |
| Accuracy Model | |  |  |  |  | |
| Accuracy | | -0.05  (0.04) | -0.21  [-0.57, 0.15] | **-0.03****  **(0.01)** | **-0.28**  **[-0.49, -0.08]** | |
| Positivity | | **0.45*****  **(0.09)** | **0.73**  **[0.45, 1.00]** | **0.51*****  **(0.01)** | **1.95**  **[1.85, 2.04]** | |
| Self-Presentational Success Model | |  |  |  |  | |
| Self-Presentational Success | | -0.07  (0.06) | -0.26  [-0.65, 0.13] | **-0.03***  **(0.01)** | **-0.23**  **[-0.43, -0.03]** | |
| Positivity | | **0.33*****  **(0.06)** | **0.72**  **[0.45, 0.99]** | **0.35*****  **(0.01)** | **1.82**  **[1.73, 1.93]** | |
| Accidental Transparency and Skilled Misrepresentation Model | |  |  |  |  |  |
| Accidental Transparency | | -0.04  (0.04) | -0.18  [-0.60, 0.25] | **-0.03****  **(0.01)** | **-0.38**  **[-0.63, -0.13]** | |
| Skilled Misrepresentation | | 0.03  (0.05) | 0.11  [-0.26, 0.47] | 0.02  (0.01) | 0.15  [-0.01, 0.35] | |
| Positivity | | **0.43*****  **(0.09)** | **0.71**  **[0.43, 1.00]** | **0.51*****  **(0.01)** | **1.99**  **[1.88, 2.10]** | |

*Swiping Outcomes of Goals, Accuracy, and Self-Presentational Success including Positivity Results*

*Note*. *** = *p* <.001, ** = *p* < .01, * *p* < .05 . We report effect size estimates, ds, for all interaction effects, calculated as a 2-standard deviation change in the moderator variable divided by the SD of the random effect estimate for the predictor in the base model (i.e., Gelman’s d) This estimate is comparable to Cohen’s *d* (see Gelman, 2008), as seen in previous work (Hater et al., 2023; Kerr et al., 2020). .

# **Table S6**

*Moderation of Expressiveness, Self-Esteem, Relationship Goals and Attractiveness With Goals, Accuracy, and Self-Presentational Success Including Positivity Result*

| Model parameters | Expressiveness | | Self-Esteem | | Relationship Goals | | Attractiveness | | Dyadic Attractiveness | |
| --- | --- | --- | --- | --- | --- | --- | --- | --- | --- | --- |
|  | *b* | *d* | *b* | *d* | *b* | *d* | *b* | *d* | *b* | *d* |
|  | (*SE*) | [95% CI] | (*SE*) | [95% CI] | (*SE*) | [95% CI] | (*SE*) | [95% CI] | (*SE*) | [95% CI] |
| Self-Presentational Goals Model |  |  |  |  |  |  |  |  |  |  |
| Accuracy | -0.02  (0.01) | -0.02  [-0.49, 0.46] | **0.06*****  **(0.01)** | **0.98**  **[0.55, 1.40]** | 0.09  (0.05) | 0.41  [-0.07, 0.90] | 0.00  (0.04) | 0.00  [-0.47, 0.47] | / | / |
| Positivity | -0.01  (0.02) | -0.28  [-0.72, 0.15] | 0.00  (0.02) | 0.03  [-0.38, 0.43] | **0.15***  **(0.08)** | **0.43**  **[0.00, 0.85]** | -0.02  (0.06) | -0.07  [-0.47, 0.34] | / | / |
| Accuracy Model |  |  |  |  |  |  |  |  |  |  |
| Accuracy | 0.00  (0.01) | 0.15  [-0.22, 0.53] | 0.00  (0.01) | 0.10  [-0.23, 0.43] | 0.02  (0.02) | 0.25  [0.22, 0.53] | -0.01  (0.01) | -0.12  [-0.49, 0.25] | **-0.01***  **(0.00)** | **-0.29**  **[-0.54, -0.04]** |
| Positivity | **0.04****  **(0.01)** | **0.51**  **[0.19, 0.83]** | 0.01  (0.01) | 0.07  [-0.24, 0.38] | **0.12****  **(0.04)** | **0.45**  **[0.14, 0.75]** | **0.14*****  **(0.03)** | **0.70**  **[0.39, 1.00]** | **0.21*****  **(0.01)** | **2.22**  **[2.11, 2.34]** |
| Self-Presentational Success Model |  |  |  |  |  |  |  |  |  |  |
| Self-Presentational Success | - | - | -0.01  (0.01) | -0.22  [-0.60, 0.16] | -0.00  (0.02) | -0.01  [-0.42, 0.40] | -0.04  (0.02) | -0.40  [-0.83, 0.02] | -0.00  (0.00) | -0.10  [0.31, 0.10] |
| Positivity | **0.02****  **(0.01)** | **0.38**  **[0.01, 0.76]** | 0.01  (0.01) | 0.18  [-0.11, 0.48] | **0.08****  **(0.03)** | **0.40**  **[0.11, 0.69]** | **0.11*****  **(0.02)** | **0.75**  **[0.46, 1.03]** | **0.14*****  **(0.00)** | **2.11**  **[2.02, 2.21]** |
| Accidental Transparency and Skilled Misrepresentation Model |  |  |  |  |  |  |  |  |  |  |
| Accidental Transparency | -0.00  (0.00) | -0.04  [-0.46, 0.38] | -0.00  (0.01) | 0.00  [-0.44, 0.44] | 0.03  (0.02) | 0.30  [-0.11, 0.70] | 0.00  (0.01) | 0.02  [-0.43, 0.46] | **-0.01*****  **(0.00)** | **-0.54**  **[-0.82, -0.26]** |
| Skilled Misrepresentation | **0.02***  **(0.01)** | **0.50**  **[0.13, 0.87]** | -0.00  (0.01) | -0.11  [-0.49, 0.27] | -0.01  (0.02) | -0.06  [-0.44, 0.32] | 0.01  (0.02) | 0.05  [-0.33, 0.44] | **0.02*****  **(0.00)** | **0.42**  **[0.21, 0.62]** |
| Positivity | **0.03****  **(0.01)** | **0.44**  **[0.11, 0.78]** | 0.01  (0.01) | 0.10  [-0.22, 0.42] | **0.10***  **(0.04)** | **0.40**  **[0.09, 0.72]** | **0.14*****  **(0.03)** | **0.70**  **[0.52, 0.87]** | **0.20*****  **(0.00)** | **2.23**  **[2.11, 2.35]** |

Note. *** = p <.001, ** = p < .01, * p < .05 . We report effect size estimates, ds, for all interaction effects, calculated as a 2-standard deviation change in the moderator variable divided by the SD of the random effect estimate for the predictor in the base model (i.e., Gelman’s d) This estimate is comparable to Cohen’s *d* (see Gelman, 2008), as seen in previous work (Hater et al., 2023; Kerr et al., 2020).

# **Table S7**

*Moderation of Attachment With Goals, Accuracy, and Self-Presentational Success Including Positivity*

| Model Parameters | Anxious Attachment | | Avoidant Attachment | |
| --- | --- | --- | --- | --- |
|  | *b* | *d* | *b* | *d* |
|  | (*SE*) | [95% CI] | (*SE*) | [95% CI] |
| Self-Presentational Goals Model |  |  |  |  |
| Accuracy | **-0.07***  **(0.03)** | **-0.54**  **[-1.00, -0.07]** | **-0.11****  **(0.03)** | **-0.77**  **[-1.23, 0.31]** |
| Positivity | 0.07  (0.04) | 0.34  [-0.07, 0.76] | -0.07  (0.05) | -0.30  [-0.72, 0.12] |
| Accuracy Model |  |  |  |  |
| Accuracy | 0.01  (0.01) | 0.17  [-0.16, 0.50] | -0.01  (0.01) | -0.16  [-0.47, 0.15] |
| Positivity | 0.00  (0.03) | 0.00  [-0.36, 0.36] | 0.01  (0.03) | 0.06  [-0.28, 0.39] |
| Self-Presentational Success Model |  |  |  |  |
| Self-Presentational Success | 0.01  (0.02) | 0.19  [-0.21, 0.60] | 0.02  (0.02) | 0.19  [-0.24, 0.62] |
| Positivity | -0.00  (0.02) | -0.04  [-0.34, 0.26] | 0.00  (0.02) | 0.02  [-0.31, 0.34] |
| Accidental Transparency and Skilled Misrepresentation Model |  |  |  |  |
| Accidental Transparency | 0.02  (0.01) | 0.32  [-0.12, 0.77] | -0.01  (0.01) | -0.18  [-0.63, 0.27] |
| Skilled Misrepresentation | -0.01  (0.01 | -0.08  [-0.46, 0.30] | 0.02  (0.02) | 0.23  [-0.17, 0.64] |
| Positivity | 0.00  (0.03) | 0.01  [-0.31, 0.34] | 0.00  (0.03) | 0.01  [-0.34, 0.33] |

Note. *** = p <.001, ** = p < .01, * p < .05 . We report effect size estimates, ds, for all interaction effects, calculated as a 2-standard deviation change in the moderator variable divided by the SD of the random effect estimate for the predictor in the base model (i.e., Gelman’s d) This estimate is comparable to Cohen’s *d* (see Gelman, 2008), as seen in previous work (Hater et al., 2023; Kerr et al., 2020).

**Table S8**

*Fixed and Random Effects of Goals, Accuracy, and Self-Presentational Success in Models where Normative Profile Was Decomposed into Normativity and Positivity.*

| Model Parameters | Fixed Effect | Target Variance |
| --- | --- | --- |
|  | *b* (*SE*) | *SD* |
| Self-Presentational Goals Model | | |
| Accuracy | 0.37*** (0.02) | **0.35***** |
| Normativity | 0.50*** (0.07) | **0.89***** |
| Positivity | 1.07*** (0.04) | **0.51***** |
| Accuracy Model |  |  |
| Accuracy | 0.04*** (0.01) | 0.08*** |
| Normativity | 0.28***(0.02) | **0.20***** |
| Positivity | 0.38*** (0.02) | **0.20***** |
| Self-Presentational Success Model |  |  |
| Self-Presentational Success | 0.04** (0.01) | **0.11***** |
| Normativity | 0.53***(0.04) | **0.43***** |
| Positivity | 0.32*** (0.01) | **0.17***** |
| Accidental Transparency and Skilled Misrepresentation Model |  |  |
| Accidental Transparency | 0.02 (0.01) | **-** |
| Skilled Misrepresentation | 0.06*** (0.01) | **0.12***** |
| Normativity | 0.31***(0.03) | **0.23***** |
| Positivity | 0.38*** (0.02) | **0.19***** |

*Note*. *** = *p* <.001, ** = *p* < .01, * *p* < .05 . Random variance in for accidental transparency was removed to resolve convergence errors in the model. SDs that met our threshold for being considered meaningful (SD > 0.10) are bolded.

# **Additional Moderators: Gender and Trait Observability**

**Did Gender Moderate Desire to be Seen or Being Seen Accurately and Positively?**

We tested if gender moderated goals to be seen accurately and positively and whether gender moderated effects of accuracy, self-presentational success, accidental transparency or skilled misrepresentation. We did not find any evidence that gender moderated wanting to be seen accurately (*b =* 0.02, *SE* = 0.06, *p* = .674) or wanting to be seen positively (*b* = 0.08, *SE* = 0.08, *p* = .291). We also did not find any evidence that gender moderated effects of accuracy (*b* = 0.01, *SE* = 0.02, *p* = .544), self-presentational success (*b* = 0.04, *SE* = 0.04, *p* = .404), accidental transparency (*b* = 0.02, *SE* = 0.02, *p* = .282), or skilled misrepresentation (*b* = 0.02, *SE* = 0.04, *p* = .591)

**Did Trait Observability Moderate Being Seen Accurately or in Line With Self-Presentational Goals?**

We tested if traits observability moderated whether targets were seen accurately or in line with their self-presentational goals. A group of 9 coders rated how observable each item was for a different study. We found that trait observability moderated accuracy (*b* = 0.01, SE = 0.01, p < .05) but not self-presentational success (*b* = 0.00, SE = 0.01, p = .451), accidental transparency (*b* = 0.01, SE = 0.01, p = .257), or skilled misrepresentation (*b* = 0.01, SE = 0.01, p = .265).

# **Table S9**

| Model | Liking | | Dyadic Liking | | |
| --- | --- | --- | --- | --- | --- |
|  | *b* | *d* | *b* | *d* | |
|  | *(SE)* | [95% CI] | *(SE)* | [95% CI] | |
| Self-Presentational Goals | | | | | |
| Accuracy | -0.01  (0.04) | -0.03  [-0.31, 0.24] | / | / | |
| Positivity | -0.03  (0.06) | -0.07  [-0.35, 0.21] | / | / | |
| Accuracy Model | | | | | |
| Accuracy | -0.01  (0.01) | -0.15  [-0.46, 0.15] | -0.00  (0.00) | 0.00  [0.00, 0.00] | |
| Positivity | **0.23*****  **(0.03)** | **1.40**  **[1.04, 1.76]** | **0.19*****  **(0.00)** | **2.71**  **[2.71, 2.71]** | |
| Self-Presentational Success Model |  |  |  |  | |
| Self-Presentational Success | -0.03  (0.02) | -0.31  [-0.70, 0.09] | -0.00  (0.00) | 0.00  [0.00, 0.00] | |
| Positivity | **0.16*****  **(0.02)** | **1.15**  **[0.87, 1.43]** | **0.19*****  **(0.00)** | **3.20**  **[3.20, 3.20]** | |
| Accidental Transparency and Skilled Misrepresentation Model | | | | |  |
| Accidental Transparency | -0.01  (0.01) | -0.15  [-0.45, 0.15] | **-0.01***  **(0.00)** | **-0.36**  **[-0.36, -0.36]** | |
| Skilled Misrepresentation | 0.01  (0.02) | 0.10  [-0.29, 0.50] | **0.01****  **(0.00)** | **0.24**  **[0.24, 0.24]** | |
| Positivity | **0.22*****  **(0.03)** | **1.41**  **[1.04, 1.79]** | **0.17*****  **(0.00)** | **2.56**  **[2.56, 2.56]** | |

*Liking Outcomes of Goals, Accuracy, Self-Presentational Success, Accidental Transparency and Skilled Misrepresentation*

*Note*. *** = *p* <.001, ** = *p* < .01, * *p* < .05 . We report effect size estimates, ds, for all interaction effects, calculated as a 2-standard deviation change in the moderator variable (swiping) divided by the SD of the random effect estimate for the predictor in the base model (i.e., Gelman’s d) This estimate is comparable to Cohen’s *d* (see Gelman, 2008), as seen in previous work (Hater et al., 2023; Kerr et al., 2020).

# **Table S10**

| Model parameters | Fixed Effect | | Target Variance | Judge Variance |
| --- | --- | --- | --- | --- |
|  | *b* (*SE*) | *SD* | | *SD* |
| Accuracy Model |  |  | |  |
| Accuracy | 0.04***(0.01) | 0.09*** | | 0.04*** |
| Positivity | 0.46***(0.03) | 0.25*** | | 0.38*** |
| Self-Presentational Success Model |  |  | |  |
| Self-Presentational Success | 0.04***(0.01) | 0.12*** | | 0.06*** |
| Positivity | 0.32***(0.02) | 0.17*** | | 0.25*** |
| Accidental Transparency and Skilled Misrepresentation Model |  |  | |  |
| Accidental Transparency | 0.01(0.01) | 0.09*** | | 0.03*** |
| Skilled Misrepresentation | 0.07***(0.01) | 0.11*** | | 0.06*** |
| Positivity | 0.43***(0.03) | 0.24*** | | 0.37*** |

*Accuracy, Self-Presentational Success, Accidental Transparency and Skilled Misrepresentation With Random Effects Modeled for Targets and Judges*

*Note*. *** = *p* <.001, ** = *p* < .01, * *p* < .05 .

**Did Physical Attractiveness Explain the Link Between Accuracy and Swiping?**

We included dyadic attractiveness (how attractive each judge uniquely found each target) as a covariate in the models testing associations between dyadic swiping and accuracy, self-presentational success, accidental transparency and skilled misrepresentation. Physical attractiveness did not explain the link between swiping and any of these variables.

# **Table S11**

*Dyadic Swiping Outcomes of Goals, Accuracy, and Self-Presentational Success With and Without Perceptions of Physical Attractiveness as a Covariate*

| Model | Dyadic Swiping Alone | | Dyadic Swiping with Attractiveness as a Covariate | | |
| --- | --- | --- | --- | --- | --- |
|  | *b* | *d* | | *b* | *d* |
|  | (*SE*) | [95% CI] | | (*SE*) | [95% CI] |
| Self-Presentational Goals Model | | | | | |
| Accuracy | / | / | | / | / |
| Positivity | / | / | | / | / |
| Accuracy Model | | | | | |
| Accuracy | **-0.03****  **(0.01)** | **-0.31**  **[-0.52, -0.11]** | | **-0.03****  **(0.01)** | **-0.35**  **[-0.58, -0.12]** |
| Positivity | **0.52*****  **(0.01)** | **1.94**  **[1.85, 2.04]** | | **0.52*****  **(0.01)** | **2.44**  **[2.35, 2.54]** |
| Self-Presentational Success Model |  |  | |  |  |
| Self-Presentational Success | **-0.03***  **(0.01)** | **-0.26**  **[-0.42, -0.09]** | | **-0.03***  **(0.01)** | **-0.24**  **[-0.39, -0.08]** |
| Positivity | **0.35*****  **(0.01)** | **1.82**  **[1.73, 1.91]** | | **0.35*****  **(0.01)** | **1.94**  **[1.83, 2.04]** |
| Accidental Transparency and Skilled Misrepresentation Model |  |  | |  |  |
| Accidental Transparency | **-0.03****  **(0.01)** | **-0.35**  **[-0.58, -0.12]** | | **-0.03****  **(0.01)** | **-0.35**  **[-0.58, -0.12]** |
| Skilled Misrepresentation | 0.02  (0.01) | 0.17  [0.00, 0.34] | | 0.02  (0.01) | 0.16  [0.00, 0.31] |
| Positivity | **0.51*****  **(0.01)** | **1.99**  **[1.88, 2.10]** | | **0.51*****  **(0.01)** | **2.52**  **[2.43, 2.62]** |

Note. *** = p <.001, ** = p < .01, * p < .05 . We report effect size estimates, ds, for all interaction effects, calculated as a 2-standard deviation change in the moderator variable (swiping) divided by the SD of the random effect estimate for the predictor in the base model (i.e., Gelman’s d) This estimate is comparable to Cohen’s d (see Gelman, 2008), as seen in previous work (Hater et al., 2023; Kerr et al., 2020).

# **Were Targets (Still) Seen in Line With Their Self-Presentational Goals in Alternate Models?**

***Were Targets Seen in Line With Their Self-Presentational Goals (when Self-Presentation is Centered on the Normative Profile of Self-Perception)?***

Our model testing whether people were seen in line with their self-presentational goals used the average desired impression to represent positivity, whereas the other models used the average self-perception. These results show the self-presentation model, where self-presentation is centered on the average self-perception instead of the average desired impression (Self-Presentation, *b* = 0.08, *SE* = 0.01, *p* < .001, *SD* = 0.11, Positivity, *b* = 0.39, *SE* = 0.02, *p* < .001, *SD* = 0.24)

***Were Targets Seen in Line With Self-Presentational Goals (When Self-Presentation is Centered on Self-Perceptions)?***

To test skilled misrepresentation and accidental transparency, we ran an additional model where targets’ self-presentation was centered on the distinctive self-perception profile, with both self-perception and positivity entered into the model. This model would reveal the extent to which judges’ impressions line up with targets’ self-presentation that is unrelated to self-views. Results of this model reveal that judges see targets in line with how targets want to be seen in ways that are different than how they see themselves (i.e., skilled misrepresentation, *b* = 0.07, *SE* = 0.01, *p* < .001). Importantly, accuracy is significant in this model (*b* = 0.08, *SE* = 0.01, *p* < .01), and it was not in the main model, indicating that accuracy here represents being seen in line with self-perceptions that overlap with desired impressions, or in other words, accuracy that is due to wanting to be seen transparently. These results confirm findings in the main text that targets have some degree of control over how they are seen.

# **Figure S1**

*Targets’ Self-Perceptions, Desired Impressions, and Judges’ Impressions of Targets on Big Five Traits With Standard Deviation Error Bars all targets (a) and five randomly selected targets (b)*

*(a)*

Score

| Item | Self-Perceptions | Self-Presentation | Judges' Impressions |
| --- | --- | --- | --- |
| TIPI1 | 4.75 | 5.32222 | 4.71932 |
| TIPI2 | 3.50562 | 2.43575 | 3.63726 |
| TIPI3 | 5.46927 | 5.44068 | 4.51084 |
| TIPI4 | 3.66667 | 1.90909 | 3.63966 |
| TIPI5 | 5.75 | 5.9162 | 4.83127 |
| TIPI6 | 4.06667 | 3.26667 | 3.94821 |
| TIPI7 | 5.61453 | 5.76836 | 4.53135 |
| TIPI8 | 2.73184 | 1.87006 | 3.47171 |
| TIPI9 | 5.05028 | 5.52222 | 4.39287 |
| TIPI10 | 2.5419 | 2.12291 | 3.47061 |

*(b)*

# **Figure S2**

*Distribution of Targets Desired Impressions*

Number of Targets

| Random Slopes of Accuracy and Positivity from Equation 1 | Accuracy | Positivity |
| --- | --- | --- |
| 0.15 | 7 |  |
| 0.25 | 26 |  |
| 0.35 | 49 |  |
| 0.45 | 49 |  |
| 0.55 | 34 |  |
| 0.65 | 12 |  |
| 0.75 | 3 |  |
| 0.85 | 0 | 7 |
| 0.95 |  | 1 |
| 1.05 |  | 6 |
| 1.15 |  | 10 |
| 1.25 |  | 16 |
| 1.35 |  | 18 |
| 1.45 |  | 35 |
| 1.55 |  | 33 |
| 1.65 |  | 26 |
| 1.75 |  | 16 |
| 1.85 |  | 10 |
| 1.95 |  | 2 |
|  |  | 0 |

*Note.* Accuracy indicates targets’ desire to be seen accurately, i.e., in line with how they see themselves. Positivity indicates targets’ desire see their positive qualities, which may reflect reality, or simply the normative, socially desirable pattern of traits.

# **Figure S3**

*Distribution of Targets’ Accuracy (a) and Self-Presentation Scores (b)*

(a)

| Accuracy Slopes | Frequency |
| --- | --- |
| -0.15 | 1 |
| -0.1 | 3 |
| -0.05 | 10 |
| 0 | 31 |
| 0.05 | 61 |
| 0.1 | 40 |
| 0.15 | 23 |
| 0.2 | 10 |
| 0.25 | 1 |
| 0.3 | 0 |
| 0.35 | 0 |
|  | 0 |

(b)

| Self-presentation Slopes |  |
| --- | --- |
| -0.15 | 0 |
| -0.1 | 6 |
| -0.05 | 18 |
| 0 | 31 |
| 0.05 | 41 |
| 0.1 | 44 |
| 0.15 | 17 |
| 0.2 | 13 |
| 0.25 | 7 |
| 0.3 | 1 |
| 0.35 | 1 |
|  | 1 |

*Note.* Accuracy slopes indicate how accurately targets were seen and self-presentation slopes indicate the extent to which targets were seen in line with their desired impression

1. The average self-perception and average desired impression were strongly correlated (*r*=.97, 95% CI [.86, .99], *p* < .001). Since other models consider positivity as the average self-perception, results of the model with the average self-perception as positivity can be found on SOM page 23.
2. Given that we are primarily interested in targets, we do not model judge variance in these models. See Table S10 for results including judge variance.
